# Development of a New Growth Standard for Breastfed Chinese Infants: What Is the Difference from the WHO Growth Standards?

**DOI:** 10.1371/journal.pone.0167816

**Published:** 2016-12-15

**Authors:** Xiaona Huang, Jenjen Chang, Weiwei Feng, Yiqun Xu, Tao Xu, He Tang, Huishan Wang, Xiaoping Pan

**Affiliations:** 1 Department of Children Health, National Center for Women and Children Health, Chinese Disease Prevention Control Center, Beijing, China; 2 Department of Epidemiology, Saint Louis University College for Public Health and Social Justice, St. Louis. MO, United States of America; 3 Department of Epidemiology, National Center for Women and Children Health, Chinese Disease Prevention Control Center, Beijing, China; TNO, NETHERLANDS

## Abstract

The objectives of this longitudinal study were to examine the trajectory of breastfed infants’ growth in China to update growth standards for early childhood, and to compare these updated Chinese growth standards with the growth standards recommended by the World Health Organization (WHO) in 2006.This longitudinal cohort study enrolled 1,840 healthy breastfed infants living in an "optimal" environment favorable to growth and followed up until one year of age from 2007 to 2010. The study subjects were recruited from 60 communities in twelve cities in China. A participating infant’s birth weight was measured within the first hour of the infant’s life, and birth length and head circumference within 24 hours after birth. Repeated weekly and monthly anthropometric measurements were also taken. Multilevel (ML) modelling via MLwiN2.25 was fitted to estimate the growth curves of weight-for-age (WFA), length-for-age (LFA), and head circumference-for-age (HFA) for the study sample as a whole and by child sex, controlling for mode of delivery, the gravidity and parity of the mother, infant’s physical measurements at birth, infant’s daily food intaking frequency per day, infant’s medical conditions, the season when the infant’s physical measurement was taken, parents’ ages, heights, and attained education, and family structure and income per month. During the first four weeks after birth, breastfed infants showed an increase in weight, length, and head circumference of 1110g, 4.9 cm, and 3.2 cm, respectively, among boys, and 980 g, 4.4 cm, and 2.8 cm, respectively, among girls. Throughout infancy, the total growth for these three was 6930 g, 26.4 cm, and 12.5 cm, respectively, among boys, and 6480 g, 25.5 cm, and 11.7 cm, respectively, among girls. As expected, there was a significant sex difference in growth during the first year. In comparison with the WHO growth standards, breastfed children in our study were heavier in weight, longer in length, and bigger in head circumference, with the exception of a few age points during the first two to four months on the upper two percentile curves.Our data suggested the growth curves for breastfed infants in China were significantly different in comparison with those based on the WHO standards. The adoption of the WHO infant growth standards among Chinese infants, as well as the methods used in the development of such growth standards in China, need careful and coordinated consideration.

## Introduction

Attained growth is the most widely used means to assess childhood growth patterns [1]. The importance of early nutrition to long term health in child development has been well documented [2]. Adequate scientific growth reference or standards are fundamental to ensure the effectiveness and reliability of childhood health assessments. Most countries, including China, have used cross-sectional surveys to develop their own growth references [3,4–8].

In recent years, growth standards developed from healthy breastfed infants have been used as a normative standard to assess the nutritional and health status of children, which has important public health implications for obesity prevention [9]. From 1997–2003, the World Health Organization (WHO) conducted a Multicentre Growth Reference Study (MGRS) to develop new growth standards for infants and young children, based on a sample of healthy breastfed infants under socio-economic conditions favorable to growth [10,11]. The new WHO Child Growth Standards (WHO standards) were released in 2006, providing an international unified tool for infant growth assessment [10,11]. The standards have been widely adopted by 125 of the 180 countries that responded to the WHO survey, but were not adopted by thirty other countries, which instead mostly preferred local standards [1]. Another 25 countries considered adopting the standards, including China [1].

Nevertheless, questions emerged regarding the applicability of the WHO standards in various countries and regions [12–14]. Roelants et al. compared the growth of breastfed children in Flanders, Belgium, with the WHO standards and found the average length of breastfed children in Flanders was close to the WHO standards values, but this did not hold for weight, BMI, or head circumference [12]. The authors concluded the use of a recent local growth reference was more appropriate for child growth monitoring in Flanders than the WHO standards [12]. Hui et al. also challenged the universal applicability of the WHO standards across all populations [14]. They reported that weight-for-age for both boys and girls in Hong Kong at most time points before three years of age was close to the 50th percentile of the WHO standards. However, the participants were shorter at three years old compared to WHO standards [14]. During the planning phase of MGRS, the WHO Working Group conducted one study to compare the growth of breastfed infants in seven countries, namely Australia, Chile, China, Guatemala, India, Nigeria, and Sweden [15]. The results suggested that breastfed babies from reasonably well-off families in different continents showed very similar growth patterns with two exceptions: Chinese infants were about 3% shorter in length at 12 months of age and Indian infants were up to 15% lighter, as compared to Australians (the reference category) [15]. Therefore, it was proposed that samples from the South and East Asia regions should be included while developing the new international growth reference. However, there were no cases from East Asia. The WHO invited China to participate in MGRS. However, the Chinese Government decided at the time not to participate in the WHO MGRS study in view of the complexity of the research methods and the fact that China performs growth surveys every ten years. Thus, the Chinese government held the belief then that MGRS was not necessary. Based on findings of the prior WHO study on infant growth, understanding whether the growth of breastfed infants in China differs from the WHO sample population was warranted. This was one of the reasons why we launched the current study.

Since the 1970s, many studies on children’s physical development have been conducted in China. For instance, national growth surveys of children under seven years were conducted in nine cities every decade [3]. The current child growth reference in China were established from the fourth survey, conducted in 2005. However, the criteria for the feeding methods used to select the study samples had not been clearly defined, a major limitation in this study, as well as most others previously conducted in China. In addition, most previous research on infant growth in China was limited by cross-sectional study design. Outdated longitudinal data from 1987 also did not account for child feeding patterns [16]. More research is needed to examine breastfed infant growth for Chinese infants.

Therefore, the objectives of this longitudinal study are: a) to describe the trajectory of breastfed infant growth for infants in China to develop a more updated growth standard for early childhood; b) to delineate the determinants of the growth trajectory; c) to understand the growth characteristics of breastfed infants in China compared to the WHO standards. The methodology of this study was largely based on the MGRS protocol [17–21].

## Methods

### Study Sites

This study was a population-based longitudinal study from Jan. 2007 to Jan. 2010. Field implementation was undertaken by the Maternal and Child Health Care Surveillance System (MCHCSS) in China, which provides postnatal care including immunization, physical examinations, and growth monitoring at no cost to infants and children from birth to seven years. The inclusion criteria for study sites were made to match the WHO criteria for living in an "optimal" environment favorable for growth [17]. The details were as follows: a) good socio-economic and nutritional status (an infant mortality rate less than 15 per 1,000 and a prevalence of less than 5 per 100 of stunting, wasting, and underweight among childhood under five years old); b) low altitude (an average altitude lower than 2000 m); c) compliance with feeding recommendations (a minimum of 20% of mothers willing to follow breastfeeding recommendations of exclusive or predominant breastfeeding for at least four months and partial breastfeeding until 12 months of age); d) strong breastfeeding support systems (e.g., hospitals with lactation consultants and breastfeeding support services); e) low population mobility (at least 80% of babies living in the community during the first years after birth); f) a surrounding environment without apparent hazards (e.g., serious microbial contamination, exposure to radiation or toxic substances); g) capacity of implementing the longitudinal study.

The selection of study sites was based on a three-stage sampling at the provincial, municipal, and community levels, separately. At the provincial level, in addition to the inclusion ctieria described above, the selection decision was made on the basis of geographic distribution (3–4 provinces were included in each of the three major geographic regions in China: the North, South, and Central regions), the integrity and technical capacity of MCHCSS, and participation interest. As a result, 11 provinces were selected among those that meet the inclusion criteria.

At the municipal level, one city per study province was chosen, often being the provincial capital city. Jingmen of Hubei was additionally selected, as it expressed high interest in this study and met the screening standards. Thus, 12 cities were enrolled. At the communities level, different numbers of representative communities from each city were selected to ensure sufficient eligible samples. Thus, a total of 60 communities from 12 cities were finally included in this study.

### Sample Enrollment

The subjects included in this study were healthy breastfed infants living in an "optimal"environment favorable for growth. The inclusion criteria for families consisted of no health-related, environmental, or economic constraints on infant growth; non-consanguineous marriage for parents; both or one of the parents having urban registration; the mother-infant pair living together, routine well-baby checkups at least up to age one; and parents willing to participate in one-year follow-up and signing of the consent form. Eligible mothers were of singleton pregnancy, a maternal age between 20 and 35 years old, had a height ≥ 1.5 m, without pregnancy complications (e.g. anemia, pregnancy induced hypertension, and diabetes), nonsmokers, and willing to deliver at designated hospitals. Only infants born to the eligible mothers with gestational age ≥ 37 completed weeks and < 42 weeks, birth weight ≥ 2500 g and < 4000 g, free of serious or life-threatening conditions at birth (e.g. congenital disease, asphyxia, birth trauma), without significant morbidity during the first year after birth (e.g. hypoxic-ischemic encephalopathy, heart disease, blood diseases, chronic nephritis, tuberculosis, chronic bronchitis, chronic diarrhea), and mothers’ compliance with feeding recommendations for this study were included.

The feeding recommendations of this study were defined as follows: 1) exclusive or predominant breastfeeding for a minimum of four months and optimally for six months of age, as suggested by national policies; 2) introduction of complementary foods between four and six months of age; and 3) continuing partial breastfeeding up to at least 12 months of age. The definitions of exclusive or predominant breastfeeding and partial breastfeeding refer to the Breastfeeding Counseling Training Course released by the WHO, as well as lactation counseling and infant feeding guidelines provided to the mothers [22]. At baseline, mothers were surveyed about their newborn’s feeding plan. Data on feeding practices were collected at each follow-up visit. Self-reported data on the infant’s one-week diet was also collected after the introduction of complementary foods.

This study was IRB approved by the Ethical Review Committee of the National Center for Women and Children's Health Chinese Center for Disease Control and Prevention. The approval number was FY2007-002. We also obtained written informed consent from parents on behalf of the children enrolled in our study.

### Anthropometric Measurements

Infants’ anthropometric measurements included three indices: weight, length, and head circumference, and were taken at birth, weekly during the first month after delivery, and monthly from two to twelve months of age. Birth weight was measured within the first hour of the infant’s life. Birth length and head circumference were measured within 24 hours after birth. Weekly and monthly measurements were taken within 23 hours of the designated infant’s age. Replicate measures were obtained for each measurement and the average values were used. The differences in replicate measures were limited to 100 g for weight and 5 mm for length and head circumference. All anthropometrists underwent the systemic standardized anthropometric training before and during the study and followed the unified anthropometry manuals. All study sites used the same high-precision measuring equipment. Regular calibration for all tools was performed daily at the community level, quarterly by urban anthropometric experts appointed for the study, and bi-annually by the National Center for Women and Children Health (NCWCH), that was in charge of this study.

### Quality Control

Quality control procedures were critical to data quality and included the following: a) construction of a longitudinal study network from national to community level, consisting of data coordination, collection, and management; quality control; and advisory for groups; b) prior to implementation, the staff from each work group underwent thorough training to standardize the protocol for anthropometric measurements, feeding methods recommendations, and questionnaire survey administration; c) pilot testing of study protocol; d) regular visits to study sites (monthly at the district level, quarterly at the city level, and semi-annually at the national level) for the calibration of measurement tools, technology capacity assessment for filed researchers who conducted the anthropometric measurements, and home and telephone interviews; e) data quality assurance through calibration of equipment, standardization of measurements and surveys, and standardizing and auditing data entry; f) routine coordination of meetings, retraining, and staff exchanges at different levels.

### Data Statistics

To accommodate the multistage-sampling and the repeated measurement data collected in this study, we conducted multilevel (ML) growth curve modelling [23] via MLwiN2.25 (http://www.bristol.ac.uk/cmm/software/mlwin/download/) to estimate the growth curves of weight-for-age (WFA), length-for-age (LFA), and head circumference-for-age (HFA) for the study sample as a whole and by child sex. This study had 16 waves of assessment data on successive cohorts from multi-communities. The community comprised the level 3 unit, subjects comprised the level 2 unit, and visiting time comprised the level 1 unit. Since the infant’s physical measurements at birth were also an important factor for the infant’s growth after birth, the multi-level model was constructed separately for the anthropometric measures taken at birth and after birth. Based on the hierarchical structure of data, a basic 2-level multivariate model with level 2 comprised of the community and level 1 comprised of the subjects, was applied to construct the Birth Physical Model (BPM), while a 3-level polynomial model was applied to construct the Growth Prediction Model After Birth (GPMAB), starting with a simple 2^nd^ degree model on age. Both the BPM and GPMAB were adjusted for the variables potentially affecting the infant’s physical growth, including the infant’s sex, mode of delivery, the gravidity and parity of mother, parents’ age, height, and attained education, and family structure and income per month, respectively. In addition to the aforementioned covariates, GPMAB also included the infant’s physical measurements at birth, infant’s daily food intaking frequency per day, infant’s medical conditions and the season when the infant’s physical measurement was taken. The final adjusted predicted model was developed based on the following criteria [23]: a) estimated value of -2*log-likelihood as small as possible; b) small level 1 variance; c) normal distributions of standardized residuals at different levels; d) stability of the variance at level 2 or 3 and f) high correlation of predicted and observed values. Based on the adjusted models’ predicted values, percentile reference values of 3rd, 15th, 50th, 85th and 97th were calculated, and the corresponding curves were depicted. We calculated the z scores relative to the WHO standards to assess whether age and sex-specific weight, length, or head circumference differed significantly from the WHO standards. All tests were two-tailed and P<0.05 was considered significant.

## Results

### Samples Description

From July 1, 2007 to July 30, 2008, 1840 (67.4%: 905 boys and 935 girls) eligible mother-infant pairs were enrolled in the study, among which 1513 (82.2%) subjects completed the one-year follow-up, and 327 (17.7%) subjects had at least one missing assessment due to scheduling conflicts (54.7%), moving out of the study sites (33.3%), child illnesses (6.1%), mother illnesses (0.9%), and unknown or other reasons (4.9%).

The demographic characteristics of 1840 subjects were presented in Table 1. Overall, approximately 61% of the average household monthly incomes were over 3000 RMB or $487 USD. The average ages of mothers and fathers were 28.0 and 30.8 years of age, respectively. On average, fathers’ heights (172.5 cm) were 12 cm more than mothers’ heights (160.5 cm). The highest educational attainment of most parents was high school or technical secondary school. Regarding the mode of delivery, 44.4% of mothers had a vaginal delivery, while 54.9% had a cesarean section. The sample characteristics between those who completed the one-year followup (i.e. compliant subjects) and those missing at least one assessment during the study period (i.e. non-compliant subjects) are comparable. Compared to the non-compliant group, parents’ highest attained education was slightly higher among the compliant group (P _Mother’s attained education_ value<0.001, P _Father’s attained education_ value<0.001).

**Table 1:** Study sample characteristics.

Demography	Total	Conpliant	Non-compliant	Comparison^a^
Mother’s age, mean±SD, t(p)	28.0±3.8	28.0±3.7	28.0±4.0	0.24(0.8096)
Mother’s height, mean±SD, t(p)	160.5±4.6	160.6±4.5	160.2±4.8	1.55(0. 1217)
Father’s age, mean±SD, t(p)	30.8±4.4	30.8±4.4	30.8±4.7	0.11(0.9092)
Father’s height, mean±SD, t(p)	172.5±5	172.6±5.1	172.1±4.8	1.47(0.1420)
Delivery(%),Chi-Square(p)				2.09(0.3521)
Vaginal delivery	44.4	45.1	41.1	
Caesarean section	54.9	54.2	58.0	
Forceps delivery	0.7	0.6	0.9	
Mother’s attained education(%), Chi-Square(p)				73.76 (<0.0001)
Primary school and under	10.8	8.4	21.1	
Junior high school	27.1	26.8	28.5	
High school(technical secondary school)	44.3	44.5	43.7	
University(college)	16.5	18.9	6.5	
Graduate and above	1.3	1.5	0.3	
Father’s attained education(%), Chi-Square(p)				66.41(<0.0001)
Primary school and under	7.7	6.0	14.7	
Junior high school	24.2	23.0	29.0	
High school(technical secondary school)	47.1	47.0	47.6	
University(college)	19.8	22.5	8.5	
Graduate and above	1.2	1.5	0.3	
Family structure(%),Chi-Square(p)				9.27(0.0259)
Nuclear family	53.8	52.8	57.8	
Consanguineous family	42.6	43.9	36.5	
Joint family	3.4	2.9	5.4	
Single parent	0.3	0.3	0.3	
Household monthly income(%),Chi-Square(p)				8.57 (0.1276)
<1000 RMB	2.4	2.2	3.4	
1000–2000 RMB	14.2	14.0	15.1	
2001–3000 RMB	22.8	22.6	23.6	
3001–5000 RMB	35.3	36.7	39.3	
5001–8000 RMB	17.8	17.3	20.2	
>8000 RMB	7.5	7.2	8.5	

a T test and Chi-Square were used to conduct the comparison on numeric variables and Categorical variables respectively between compliant and non-compliant group.

### Breastfed Infants’ Growth Centile Curves from a Chinese Population

**Fig 1:**
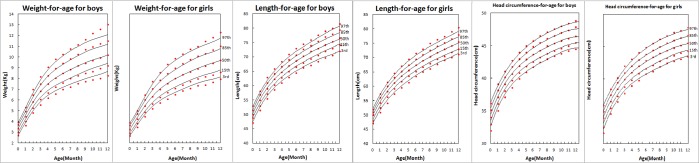
Predicted percentile curves derived from the adjusted multivariable models represented with lines and crude measured values with dots: weight-, length-, and head circumference-for-age for boys and girls from birth to 12 months. From top to bottom is 97th, 85th, 50th, 15th, and 3rd.

Fig 1 illustrated the estimated percentile curves derived from the adjusted multivariable models (Tables 2 and 3) and the crude measured values for these three indices for boys and girls from birth to 12 months. Our adjusted predicted models and the crude measured values for the three outcomes of interest were all highly correlated (rr>0.99). The estimated results demonstrated satisfactory goodness of fit (Fig 1).

**Table 2:** BMP adjusted predicted model for weight, length, and head circumference at birth.

Parameters	Birth Weight		Birth Length		Birth Head Circumference	
	Estimate(s.e.)^a^	*p*	Estimate(s.e.)^b^	*p*	Estimate(s.e.)^b^	*p*
			**Fixed effects**			
Intercept	-3.421(0.354)	<0.001	-13.886(1.447)	<0.001	-6.351(1.115)	<0.001
Sex = Boy	0.007(0.015)	<0.001	0.401(0.061)	<0.001	0.334(0.047)	<0.001
Sex = Girl	Reference					
Mode of delivery = Cesarean section					0.306(0.05)	<0.001
Mode of delivery = Vaginal births	Reference					
Motherage	0.006(0.002)	0.009				
Motherheight	0.012(0.002)	<0.001	0.049(0.007)	<0.001	0.021(0.006)	0.000
Mother education = High school			-0.291(0.082)	0.000		
Mother education = Bachelor and over			0.02(0.131)	0.882		
Mother education = Junior high school and under						Reference
Fatherheight	0.008(0.002)	<0.001	0.034(0.007)	<0.001	0.016(0.005)	0.002
Father education = High school			0.214(0.083)	0.010	-0.048(0.055)	0.387
Father education = Bachelor and over			0.002(0.126)	1.000	-0.157(0.078)	0.043
Mother education = Junior high school and under						Reference
**Random effects (variance components)**						
Level 2(community)^**b**^	0.003(0.001)	<0.001	0.165(0.043)	<0.001	0.141(0.034)	<0.001
Level 1(subject)	0.1(0.003)	0.014	1.625(0.055)	0.000	0.94(0.032)	<0.001
-2*LL*(IGLS)^**c**^	995.897		5996.419		4928.687	

a Standard errors in brackets.

b, As results shown, all the random coefficients were statistically significant (P_Wald test_ <0.05), indicating that infants’ physical status at birth presented significantly different from different communities.

c -2*loglikelihood (Iterative Generalised Least Squares (IGLS) Deviance).

**Table 3:** Final GPMAP adjusted predicted model for breastfed infants’ weight-for-age, length-for-age, and head circumference-for-age, respectively, as a fourth fixed and second degree polynomial on age.

Parameters	Weight-for-age		Length-for-ge		Head circumference-for-age	
	*H*_0_ = 4, *H*_1_ = 2^a^		*H*_0_ = 4, *H*_1_ = 2^a^		*H*_0_ = 4, *H*_1_ = 2^a^	
	Estimate(s.e.)^b^	*p*	Estimate(s.e.)^b^	*p*	Estimate(s.e.)^b^	*p*
**Fixed effects**						
Intercept	-5.228(0.294)	<0.001	-40.019 (1.402)	<0.001	-22.255(0.659)	<0.001
Age	-0.004(0.001)	<0.001	-0.063(0.007)	<0.001	-0.007(0.001)	<0.001
Age2	0.000(0.000)	<0.001	0.000(0.000)	<0.001	0.000(0.000)	<0.001
Age3	0.000(0.000)	<0.001	0.000(0.000)	0.001	0.000(0.000)	<0.001
Age4	0.000(0.000)	<0.001			0.000(0.000)	0.001
Sex = Boy	-0.072(0.014)	<0.001			0.099(0.032)	0.002
Sex = Girl						Reference
Sex = Boy×Age	0.009(0.001)	<0.001	0.014(0.001)	<0.001	0.009(0.001)	<0.001
Sex = Boy×Age2	0.000(0.000)	<0.001	0.000(0.000)	<0.001	0.000(0.000)	<0.001
Sex = Boy×Age3	0.000(0.000)	<0.001	0.000(0.000)	0.000	0.000(0.000)	0.002
Sex = Boy×Age4	0.000(0.000)	<0.001	0.000(0.000)	0.005	0.000(0.000)	0.022
Disease = With disease	-0.059(0.005)	<0.001	-0.046(0.017)	0.007		
Disease = Without disease						Reference
Season = Summer	0.009(0.010)	0.346	0.132(0.033)	<0.001		
Season = Autumn	0.023(0.011)	0.041	0.303(0.039)	<0.001		
Season = Winter	0.014(0.01)	0.166	0.168(0.035)	<0.001		
Season = Spring						Reference
Season = Summer×Age	0.000(0.000)	0	0.000(0.000)	0.013		
Season = Autumn×Age	0.000(0.000)	<0.001	-0.001(0.000)	0.000		
Season = Winter×Age	0.000(0.000)	0.017	0.000(0.000)	0.021		
Birthweight	0.792(0.021)	<0.001	1.04(0.091)	<0.001	0.395(0.056)	<0.001
Birthlength	0.028(0.005)	<0.001	0.555(0.021)	<0.001	0.033(0.013)	0.010
Birthhead	0.025(0.006)	<0.001	0.075(0.025)	0.003	0.568(0.015)	<0.001
Gravida = Non-first pregnancy	0.031(0.014)	0.031				
Gravida = First pregnancy						Reference
Gravida = Non-first pregnancy×age	-0.001(0.000)	0.000				
Parity = Non-first birth	0.056(0.025)	0.028				
Parity = First birth						Reference
Mode of delivery = Cesarean section	-0.026(0.011)	0.014				
Mode of delivery = Vaginal births						Reference
Motherage	-0.003(0.002)	0.063				
Motherheight			0.019(0.005)	0.000		
Motherheight×Age			0.000(0.000)	<0.001		
Mother education = High school	-0.013(0.012)	0.249	0.055(0.048)	0.256		
Mother education = Bachelor and over	0.003(0.017)	0.862	0.159(0.073)	0.030		
Mother education = Junior high school and under						Reference
Mother education = High school×Age					0.001(0.000)	0.001
Mother education = Bachelor and over×Age					0.001(0.000)	0.001
Fatherheight	0.003(0.001)	0.005	0.018(0.005)	0.000		
Fatherheight×Age			0.000(0.000)	<0.001		
Formula	0.005(0.002)	0.033	0.025(0.008)	0.001		
Cereal					-0.017(0.006)	0.003
Meat			-0.049(0.016)	0.002		
Bean	0.026(0.013)	0.045				
Vegetable	0.011(0.004)	0.017	0.030(0.014)	0.030		
Fruit	0.014(0.005)	0.004				
**Random Effects**						
Level 3(community) ^**c**^						
σ^2^_*ν*0_	0.003(0.001)	0.001	0.189(0.042)	<0.001	0.055(0.013)	<0.001
Level 2(infant) ^**d**^						
σ^2^_*μ*0_	0.037(0.002)	<0.001	0.664(0.029)	<0.001	0.28(0.012)	<0.001
σ_*μ*10_	-0.001(0.000)	<0.001	-0.003(0.000)	<0.001	-0.001(0.000)	<0.001
σ^2^_*μ*1_	0.000(0.000)	<0.001	0.000(0.000)	<0.001	0.000(0.000)	<0.001
σ_*μ*20_	0.000(0.000)	<0.001	0.000(0.000)	<0.001	0.000(0.000)	<0.001
σ_*μ*12_	0.000(0.000)	<0.001	0.000(0.000)	<0.001	0.000(0.000)	<0.001
σ^2^_*μ*2_	0.000(0.000)	<0.001	0.000(0.000)	<0.001	0.000(0.000)	<0.001
Level 1(visit)						
σ^2^_*e*0_	0.048(0.001)	<0.001	0.532(0.006)	<0.001	0.162(0.002)	<0.001
-2*LL*(IGLS)^e^	8751.320		60100.724		344442.472	

a H0 for fixed effects degree; H1 for random effects degree

b Standard errors in brackets

c σ^2^_*ν*0_ presented the random effects on the Level 3. As results shown, all the random coefficients were statistically significant (P_Wald test_ <0.05), indicating that infants growth pattern living in different communities presented significantly different.

d σ^2^_*μ*0_,σ^2^_*μ*1_and σ^2^_*μ*2_ presented the random effects on the Level 2. As results shown, all the random coefficients were statistically significant (P_Wald test_ <0.05), indicating that the different individuals’ corresponding growth indicator changing with time were significant different.

e -2*loglikelihood (Iterative Generalised Least Squares (IGLS) Deviance).

It is noteworthy that on 97th percentile curve of WFA, the difference between the raw data and the predicted value became larger after 10 months old, which may be due to the degree of dispersion in our data. It appears that the older age a child was, the greater the degree of dispersion was observed for WFA, compared to LFA or HFA.

**Fig 2:**
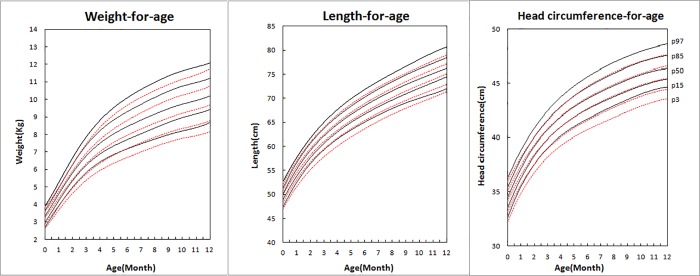
Difference between girls and boys for 3rd, 15th, 50th, 85th, and 97th predicted percentile values: weight-, length-, and head circumference-for-age for boys and girls from birth to 12 months. Boys’ predicted percentile curves are represented with solid lines and girls’ curves with dotted lines. From top to bottom is 97th, 85th, 50th, 15th, and 3^rd^ percentile.

We further stratified our analysis by child sex. As expected, there was a significant sex difference in growth during the first year (P _child sex_ value <0.0001, Tables 2 and 3). In Fig 2, based on the 50th percentile curve, birth weight among boys was on average 90 g more than birth weight among girls. Similarly, length and head circumference among boys were on average 0.2 cm greater than among girls. Between one and twelve months, the growth advantages for boys remained and the differences by child sex expanded to 240–530 g, 0.7–1.4 cm, and 0.6–1.0 cm by the end of the study followup. The gap between boys and girls was comparable in different percentile curves and seemed to widen with increasing age, especially during the first six months after birth (P _child age x sex_ value<0.0001, Table 3).

The growth medians and the corresponding standard deviations were also detailed in Table 4**.** The standard deviation was an estimate of the SD curve, given by the MLWin software. During the first four weeks after birth, breastfed infants showed an increase in weight, length, and head circumference of 1110 g, 4.9 cm, and 3.2 cm, respectively, among boys, and 980 g, 4.4 cm, and 2.8 cm, respectively, among girls. Throughout infancy, the total growth for these three indices was 6930 g, 26.4 cm, and 12.5 cm, respectively, among boys and 6480 g, 25.5 cm, and 11.7 cm, respectively, among girls.

**Table 4:** Breastfed infants’ growth reference median values and standard deviation for Chinese population from birth to 12 months old.

Age	Boys						Girls					
	Weight-for-age (kg)		Length-for-age (cm)		Head circumference-for-age (cm)		Weight-for-age (kg)		Length-for-age (cm)		Head circumference-for-age (cm)	
	Median	SD	Median	SD	Median	SD	Median	SD	Median	SD	Median	SD
Birth	3.36	0.32	50.1	1.3	34.1	1.0	3.26	0.32	49.9	1.3	33.9	1.0
Week 1	3.39	0.34	51.1	1.4	34.8	0.9	3.30	0.34	50.8	1.3	34.4	0.9
Week 2	3.73	0.34	52.4	1.4	35.7	0.9	3.58	0.34	51.9	1.3	35.2	0.9
Week 3	4.11	0.34	53.6	1.4	36.5	0.9	3.90	0.35	53.1	1.4	35.9	0.9
Week 4	4.47	0.36	55.0	1.4	37.3	0.9	4.24	0.35	54.3	1.3	36.7	0.9
month 2	5.86	0.44	59.1	1.4	39.2	0.9	5.49	0.45	58.2	1.4	38.5	0.9
Month 3	6.79	0.54	62.4	1.5	40.6	0.9	6.35	0.56	61.2	1.5	39.8	0.9
Month 4	7.51	0.64	64.7	1.6	41.9	0.9	7.04	0.66	63.5	1.6	41.0	0.9
Month 5	8.05	0.73	66.8	1.7	42.8	1.0	7.53	0.75	65.5	1.7	41.9	1.0
Month 6	8.51	0.78	68.7	1.8	43.7	1.0	8.04	0.80	67.4	1.8	42.8	1.0
Month 7	8.86	0.82	70.2	1.8	44.4	1.1	8.36	0.84	68.9	1.8	43.4	1.1
Month 8	9.21	0.86	71.8	1.9	44.9	1.1	8.68	0.88	70.4	1.9	43.9	1.1
Month 9	9.48	0.90	73.0	2.0	45.4	1.1	8.95	0.89	71.7	1.9	44.5	1.1
Month 10	9.76	0.90	74.2	2.0	45.7	1.0	9.25	0.92	72.9	2.0	44.8	1.0
Month 11	9.98	0.91	75.3	2.1	46.1	1.0	9.48	0.94	74.1	2.0	45.2	1.1
Month 12	10.29	0.91	76.5	2.1	46.6	1.0	9.74	0.96	75.4	2.0	45.6	1.0

### Comparison with WHO Growth Standards

**Fig 3:**
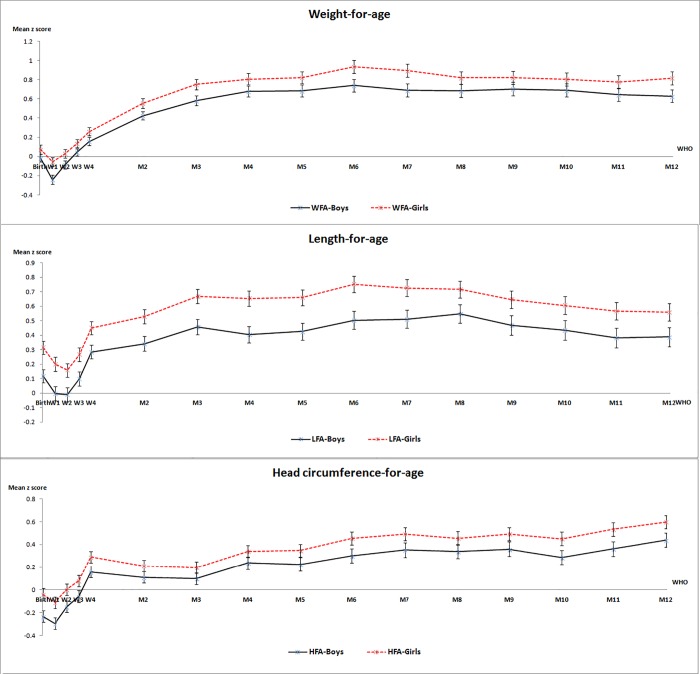
The mean z score of weight, length, and head circumference, with reference to the WHO growth standards. Boys’ mean z score represented with solid lines, and girls’ with dotted lines. Most of the z values were statistically significant, except the six age points: birth for WFA-Boys; W2 for WFA-Gilds; W1 and W2 for LFA-Boys; birth and W2 for HFA-Girls.

In comparison with the WHO growth standards [10], Fig 3 showed that boys’ weight during the first two weeks after birth and girls’ at one week after birth in our study was lower than the WHO standards, with mean z scores of -0.24, -0.08 and -0.05, respectively (P value <0.05). For the remainder of the followup visits till age one, WFA in both sexs in our study was higher than the WHO standards (P value <0.05) and most of the mean z scores were over 0.7 (Fig 3). In addition, both boys’ and girls’ birth length were higher than that of the WHO standards [10], with mean z scores of 0.12 and 0.31 (P value <0.0001), respectively. And this trend remained until 12 months after birth with mean z scores of 0.28–0.55 and 0.16–0.75 (P value <0.05), respectively, while boys’ length at one and two weeks after birth were not significantly different from WHO standards. In girls, 70% of age points had z scores over 0.5 during the first whole year (Fig 3).

Compared to the WHO standards [11], boys’ birth head circumference of the breastfed infant from our study was lower with mean z scores of -0.24, and this situation continued until three weeks after birth (P value<0.05). However, subsequent boys’ head circumference exceeded the WHO standard with mean z scores of 0.10–0.44 (P value<0.05). To the contrary, girls’ head circumference at birth and from two weeks after birth had no significant difference from the WHO standard, but was slightly lower at one week after birth with mean z scores of -0.11 (P value<0.05). From three weeks after birth until 12 months old, girls’ head circumference was also greater than the WHO standard, with mean z scores of 0.08–0.60 (P value<0.05). It is also noteworthy that the z scores of 70% of the girls’ head circumference during study followup assessment were over 0.30 (Fig 3).

**Fig 4:**
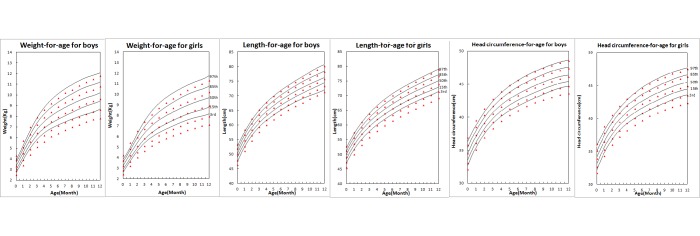
Comparisons of weight-, length-, and head circumference-for- age percentile curves from this study and WHO by child sex from birth to 12 months. Findings fom this study were represented with lines and WHO with triangle signs. From top to bottom represents the 97th, 85th, 50th, 15th, and 3rd percentiles.

We further compared the growth percentile curves of WFA, LFA, and HFA by child sex from the present study to the WHO standard percentile curves. As Fig 4 shows, the shapes of the two sets of curves were very similar, but the growth percentile curve estimates by the two groups varied on different percentile curves. Similar to the comparisons on the median level between the two studies (Fig 3), breastfed infants in our study were heavier in weight, longer in length, and bigger in head circumference than those in MGRS, with the exception of a few age points during the first two to four months on the upper two percentile curves.

The boys and girls included in our study fell below the WHO standards by 60–400 g and 270–340 g, respectively, during the first three months after birth, but were above the WHO standards by 210–470 g and 300–510 g, respectively, for the remaining study period until 12 months of age, when the corresponding values of the WHO standard approximately equaled the predicted 91st-93rd values of the present study. Notable differences were also found in the 15th and 3rd percentile curves. The values of the 15th curve of boys’ WFA or LFA in the WHO Standards were close to the corresponding values of our 1st-3rd or 3rd-8th percentile curves, respectively, and those of the 3rd curve were below the lowest limit of our populations. Similar results were also evident for the girls group.

Consequently, during the first year of life, estimates of obesity (with the 97th percentile of weight-for-age as the cut-off point) for Chinese infants were higher when based on the standard draw from the WHO MGRS, while underweight (with the 3rd percentile of weight-for-age as the cut-off point), and stunting (with the 3rd percentile of length-for-age as the cut-off point) were lower.

### Comparison with growth reference constructed by the 2005 nine-city study

Compared to the infant growth reference from the 2005 nine-city study (3), the growth levels reached by breastfed infants on the 97th percentile curve in our study were relatively lower both for weight and length. The differences between the two studies across the first year were 280–560 g and 1.1–1.4 cm for boys and 10–370 g and 0.7–1.5 cm for girls, respectively. However, conflicting results occurred on the 50th curve, as well as the lower two percentile curves. Especially on the 3rd percentile curve, the boys’ weights and lengths in our study were 120–630 g and 1.2–1.9 cm above the current national reference, which also presented in the girls group.

## Discussion

The present study was the first longitudinal study of breastfed infants based on a large sample of Chinese children. Compared to Chinese growth standards developed 25 years ago by means of a longitudinal study design [16], a positive secular trend in infant growth was observed in the present study during the first year of life, notably among girls. Specifically, the median weight, length, and head circumference have increased by an average of 150 g, 0.5 cm, and 0.2 cm, respectively, among boys, and 220 g, 0.9 cm, and 0.3 cm among girls. These increases in infant growth were likely a result of improved living conditions and economic development. Similar increases in infant anthropometric measurements were observed in another widely adopted Chinese growth standard, which was based on a large cross-sectional study of Chinese infants from nine cities in China in 2005 [3, 24]. This positive secular trend in infant growth between our findings and those from the aforementioned cross-sectional study in 2005 of Chinese infant growth highlights the importance of updating the growth standards of breastfed children.

The considerable divergence in infant growth between our study and the 2005 nine-cities study, as noted above, likely resulted from the differences in study design and the characteristics of study samples, specifically from the differences in the types of feeding methods for the enrolled subjects. In the nine- cities study, 36.3% of the study sample reported partially breast-feeding during the first four months and 15.1% exclusively formula-feeding. Only 48.6% of infants were exclusively or predominantly breastfeeding [25], whereas the present study was based solely on breastfed infants. The dramatic departure of the 97th percentile growth curve developed in the nine- cities survey was likely a consequence of the introduction of formula-fed infants, who might represent the majority of those in the upper percentiles. Previous studies have observed that infants who were exclusively or predominantly breast-fed for the first 4–6 mo, and partially breastfed thereafter, experienced more rapid growth in the first 2–3 months of life but this difference in growth rate by feeding types appeared to taper off thereafter, in relation to growth reference developed based on formula-fed infants[26, 27].Differences in feeding types were also likely to contribute to the different growth patterns in the lower percentiles between the two studies, particularly for the 3rd percentile curves. Infants in the current study followed the recommended feeding practices with high-quality complementary diets. To the contrary, about 11% of infants in the nine- cities study introduced complementary diets before three months of age and 82.5% between four and five months after birth [25]. The higher probability of inappropriate feeding behavior was speculated to move the lower centile curves of the nine- cities reference downwards by one standard deviation. To sum up, the dramatic difference from our study findings in the 97th and 3rd percentile curves probably rendered the growth curves developed by the nine- cities study inadequate for monitoring obesity/overweight and underweight or stunting during the first year after birth. In contrast, the current study’s curves can facilitate the assessment of populations with high rates of over-nutrition or malnutrition.

An updated infant growth standard based on data from longitudinal studies from a study sample meeting the recommended feeding practices is critical to provide an appropriate reference for the optimal growth and development of healthy Chinese children during their first year of life. In comparison with the growth curves of the WHO standards [10, 11], it was evident based on findings from this study that breastfed infants in China were heavier and longer on average than the infants represented in the WHO standards. This applied to all assessment intervals during the first year of our study, except for infants zero to three months on the upper two centiles, whereby infants in China were somewhat lower in weight and shorter in length. These differences may be the result of genetic variations, exposured to vastly different environmental factors, and differences in socioeconomic status [15, 28]. In addition, despite a similar study design and inclusion critiera to the WHO MGRS study, we limited the inclusion criteria of altitude to 2000 meters, which was relatively higher than the definition of 1500 meters proposed in WHO MGRS. Previous studies have reported that the growth of children living at relatively high altitude might be lower than those living in areas of lower altitude [29,30]. Nevertheless, there were only 70 infants in our study (3.8%) from the city of Yuxi, which has an altitude of 1640 meters.

Noteworthy, the weight and length values of Chinese infants from this study were shown to be slightly lower at birth (average differences of 220 g and 0.69 cm among boys and 190 g and 0.19 cm among girls) as compared to the reference issued in Sweden [31], but were higher than that of the Swedish population until 12 months of age, among whom the maximum difference in length was 1.12 cm. Similar results were obtained when comparing weight and length values of Chinese infants from this study to Belgian reference [7]. These findings demonstrate the growth potential of Chinese infants did not lag behind that seen in industrialized countries when early nutrition among healthy Chinese infants followed breastfeeding recommendations.

In view of these findings, the adoption of the WHO infant growth standards in China would result in an increased number of Chinese infants classified as overweight or obese but a decreased number of infants classified as underweight or stunted. With a vast territory, significant regional differences in child development exist in China. In urban areas, where obesity has become the main public health problem, the WHO standards might be helpful to avoid excess weight gain but probably will lead to inappropriate assessment concerning weight-for-age, resulting in needless parental worrying. By contrast, in rural areas, especially in Western China, malnutrition is still the most common health problem adversely affecting child growth. Quite a few malnourished infants would be classified as having normal development by the infant growth standard based on the WHO MGRS study and subsequently miss out on early interventions.

Similar results were reported from Juliusson et al. on Norwegian and Belgian children that the number of Belgian and Norwegian children below -2 SD lines of the WHO standards was lower and above +2 SD higher than expected [32]. Lately, Natale et al. based on the systematic review from studies performed in 55 countries or ethnic groups, found that weight, height and head circumference varied somewhat among different national and ethnic groups, especially for head circumference. When compared with the MGRS means, the means for head circumference in many groups were consistently 0.5–1 SD above. Thus this study indicated that using the WHO charts would put many children at risk for misdiagnosis of macrocephaly or microcephaly, and should be cautious to use one single international standards for head circumference for different ethic or countries [33].

The strengths of the present study include its prospective design with repeated measures on breastfeeding infant growth. Most previous research on infant growth was limited by cross-sectional study design and without the clear definition of infant feeding methods [34,35]. Our data should also be interpreted with caution. We strongly recommended enrolled parents to exclusive breastfeeding for at least for 4 months, and continued partial breastfeeding up to at least 12 months. However, 18% of the study sample did not complete the one year follow-up. As **Table 1**showed, as compared to the non-compliant group, parents’ highest attained education was a bit higher among the compliant group. Our study findings may have overestimated the average infant growth of breastfeeding infants due to the missing anthropometric data of non-compliant samples [36]. However, the non-compliant rate was lower than our estimate of 20%.

In conclusion, our study was a large, longitudinal-based study of a sample of healthy breastfed Chinese infants. This study showed that the growth curves for breastfed infants in China were significantly different in comparison with those based on the WHO MGRS study, as well as those from the current national growth standards based on the Chinese nine-cities study. The adoption of the WHO infant growth standards among Chinese infants, as well as the methods used in the development of such growth standards in China, need careful and coordinated consideration.

## References

[1] de Onis M, Onyango A, Borghi E, Siyam A, Blössner M, Lutter C; WHO Multicentre Growth Reference Study Group. Worldwide implementation of the WHO Child Growth Standards. Public Health Nutr. 2012; 15:1603–10. 10.1017/S136898001200105X 22717390

[2] Lillycrop KA, Burdge GC. Epigenetic mechanisms linking early nutrition to long term health. Best Pract Res Clin Endocrinol Metab. 2012; 26:667–76. 10.1016/j.beem.2012.03.009 22980048

[3] Zong XN, Li H. Constrcuction of a new growth references for China based on urban Chinese children: comparison with the WHO growth standards. Plos One. 2013; 8(3): e59569 10.1371/journal.pone.0059569 23527219 PMC3602372

[4] Marwaha RK, Tandon N, Ganie MA, Kanwar R, Shivaprasad C, Sabharwal A, et al Nationwide reference data for height, weight and body mass index of Indian schoolchildren. Natl Med J India. 2011; 24:269–77. 22680077

[5] Elmali F, Altunay C, Mazicioglu MM, Kondolot M, Ozturk A, Kurtoglu S. Head circumference growth reference charts for Turkish children aged 0–84 months. Pediatr Neurol. 2012;46:307–11. 10.1016/j.pediatrneurol.2012.02.016 22520352

[6] Del-Rio-Navarro BE, Velazquez-Monroy O, Santos-Preciado JI, Lara-Esqueda A, Berber A, Loredo-Abdala A, et al Mexican anthropometric percentiles for ages 10–18. Eur J Clin Nutr. 2007;61:963–75. 10.1038/sj.ejcn.1602612 17228343

[7] Roelants M, Hauspie R, Hoppenbrouwers K.References for growth and pubertal development from birth to 21 years in Flanders, Belgium. Ann Hum Biol. 2009;36:680–94. 10.3109/03014460903049074 19919503

[8] Saari A, Sankilampi U, Hannila ML, Kiviniemi V, Kesseli K, Dunkel L. New Finnish growth references for children and adolescents aged 0 to 20 years: Length/height-for-age, weight-for-length/height, and body mass index-for-age. Ann Med. 2011; 43:235–48. 10.3109/07853890.2010.515603 20854213

[9] Garza C, de Onis M. Rationale for developing a new international growth reference. Food Nutr Bull. 2004; 25(1 Suppl): S5–14. 15069915 10.1177/15648265040251S102

[10] WHO Multicentre Growth Reference Study Group. WHO Child Growth Standards: Length/height-for-age, weight-for-age, weight-for-length, weight-for-height, and body mass index-for-age: Methods and development Geneva: World Health Organization, 2006.

[11] WHO Multicentre Growth Reference Study Group. WHO Child Growth Standards: Head circumference–for- age, arm circumference-for-age, triceps skinfold-for-age and subscapular skinfold-for-age: Methods and development Geneva: World Health Organization, 2007

[12] Roelants M, Hauspie R, Hoppenbrouwers K. Breastfeeding, growth and growth standards: Performance of the WHO growth standards for monitoring growth of Belgian children. Ann Hum Biol. 2010;37:2–9. 10.3109/03014460903458080 19968593

[13] Wright C, Lakshman R, Emmett P, Ong KK. Implications of adopting the WHO 2006 Child Growth Standard in the UK: two prospective cohort studies. Arch Dis Child. 2008; 93:566–9. 10.1136/adc.2007.126854 17908712 PMC2532956

[14] Hui LL, Schooling CM, Cowling BJ, Leung SS, Lam TH, Leung GM. Are universal standards for optimal infant growth appropriate? Evidence from a Hong Kong Chinese birth cohort. Arch Dis Child. 2008; 93:561–5. 10.1136/adc.2007.119826 17556396

[15] WHO Working Group on the Growth Reference Protocol and WHO Task Force on Methods for the Natural Regulation of Fertility. Growth patterns of breastfed infants in seven countries. Acta Paediatr. 2000; 89: 215–22. 10709894 10.1080/080352500750028861

[16] Ding ZY, Lu XY, Wu YH, Wang HS, Liu QH, Zhang JG. Longitudinal observation of children growth from birth to 72 months of age. Chin J Pediatr. 1996; 34:93–97.

[17] de Onis M, Garza C, Victora CG, Onyango AW, Frongillo EA, Martines J. The WHO Multicentre Growth Reference Study: planning, study design, and methodology. Food Nutr Bull. 2004; 25(1 Suppl):S15–26. 15069916 10.1177/15648265040251S103

[18] de Onis M, Onyango AW, Van den Broeck J, Chumlea WC, Martorell R. Measurement and standardization protocols for anthropometry used in the construction of a new international growth reference. Food Nutr Bull. 2004; 25(1 Suppl):S27–36. 15069917 10.1177/15648265040251S104

[19] WHO Multicentre Growth Reference Study Group. Complementary feeding in the WHO Multicentre Growth Reference Study. Acta Paediatr Suppl. 2006; 450:27–37. 16817676 10.1111/j.1651-2227.2006.tb02373.x

[20] WHO Multicentre Growth Reference Study Group. Breastfeeding in the WHO Multicentre Growth Reference Study. Acta Paediatr Suppl. 2006; 450:16–26. 16817675 10.1111/j.1651-2227.2006.tb02372.x

[21] WHO Multicentre Growth Reference Study Group. Enrolment and baseline characteristics in the WHO Multicentre Growth Reference Study. Acta Paediatr Suppl. 2006; 450:7–15. 16817674 10.1111/j.1651-2227.2006.tb02371.x

[22] World Health Organization, UNICEF, editor. Translated by Division of Maternal and Child Health of the Ministry of Health of the People's Republic of China Breastfeeding counseling: A training course (student handbook). Beijing: Beijing Medical University and Peking Union Medical College Press, 1997:1.

[23] Goldstein H (1986). Efficient statistical modelling of longitudinal data. Annals of Human Biology. 13:129–141 3707042 10.1080/03014468600008271

[24] Coordinating study group of nine cities on the physical growth and development of children. A national survey on growth of children under 7 years of age in nine cities of china, 2005. Chin J Pediatr. 2007; 45:609–14 18021536

[25] Division of Maternal and Child Health of the Ministry of Health of the People's Republic of China, Coordinating study group of nine cities on the physical growth and development of children, Capital Institute of Pediatrics, edition. The national growth survey of children under 7 years in the nine cities of china. People’s medical publishing house. 2008.

[26] Victora CG, Morris SS, Barros FC, de Onis M, Yip R.The NCHS Reference and the growth of breast- and bottle-fed infants, J Nutr. 1998; 128: 1134–8. 9649596 10.1093/jn/128.7.1134

[27] de Onis M, Onyango AW. The Centers for Disease Control and Prevention 2000 growth charts and the growth of breastfed infants. Acta Paediatric. 2003;92:413–9. 10.1111/j.1651-2227.2003.tb00570.x 12801105

[28] Towne B, Demerath EW, Czerwinski SA. The genetic epidemiology of growth and development In: Cameron N (ed) Human growth and development. London, Academic, 2006;103–137.

[29] Argnani L, Cogo A, Gualdi-Russo E. Growth and nutritional status of Tibetan children at high altitude. Coll Antropol. 2008;32:807–12. 18982755

[30] Malkoç I, Mazıcıoğlu MM, Özkan B, Kondolot M, Kurtoğlu S, Yeşilyurt H. Height, weight and body mass index percentiles of children aged 6–14 years living at moderate altitudes. J Clin Res Pediatr Endocrinol. 2012;4:14–20. 10.4274/jcrpe.559 22394700 PMC3316457

[31] Wikland KA, Luo ZC, Niklasson A, Karlberg J.Swedish population-based longitudinal reference values from birth to 18 years of age for height, weight and head circumference. Acta Paediatr. 2002;91:739–54. 12200898 10.1080/08035250213216

[32] Júlíusson PB, Roelants M, Hoppenbrouwers K, Hauspie R, Bjerknes R. Growth of Belgian and Norwegian children compared to the WHO growth standards: prevalence below -2 and above +2 SD and the effect of breastfeeding. Arch Dis Child. 2011; 96:916–21. 10.1136/adc.2009.166157 19948662

[33] Natale V, Rajagopalan A.Worldwide variation in human growth and the World Health Organization growth standards: a systematic review. BMJ Open. 2014; 8:4(1). 10.1136/bmjopen-2013-003735 PMC3902406 24401723

[34] Rosario AS, Schienkiewitz A, Neuhauser H.German height references for children aged 0 to under 18 years compared to WHO and CDC growth charts.Ann Hum Biol.2011; 8:121–130. 10.3109/03014460.2010.521193 20939749

[35] Freeman JV, Cole TJ, Chinn S, et al Cross sectional stature and weight reference curves for the UK,1990.Arch Dis Child.1995;73:17–24. 7639543 10.1136/adc.73.1.17 PMC1511167

[36] Chen CM, He W, Chang SY. The changes of the attributable factors of child growth. Journal of Hygiene Research. 2006;35:765–68. 17290762

